# Pulmonary hypertension in the developing world: Local registries, challenges, and ways to move forward

**DOI:** 10.21542/gcsp.2020.14

**Published:** 2020-04-30

**Authors:** Majdy Idrees, Ghazwan Butrous, Ana Mocumbi, Bhagavathula Sastry, Ahmed Ibrahim, Khalid Alobaidallah, Ahmed Hassan, Ayman A.H. Farghaly, Magdi Yacoub

**Affiliations:** 1 Division of Pulmonary Medicine, Prince Sultan Military Medical City, Riyadh, Saudi Arabia; 2 School of Pharmacy, University of Kent, Canterbury, UK; 3 Instituto Nacional de Saúde Vila de Marracuene, Maputo, Mozambique; 4 Department of Cardiology Science, Care Hospitals, Hyderabad, India; 5 Aswan Heart Center, Aswan, Egypt; 6 Cardiology Department, Cairo University, Cairo, Egypt; 7 Department of Pulmonary Medicine, Military Respiratory Center, Cairo, Egypt

## Introduction

Pulmonary hypertension (PH) is a disease that can only be appropriately managed in the ‘rich’ developed countries, as both diagnostic and therapeutic interventions are extremely expensive and expectations for these to be adopted by the developing, economically-challenged countries are neither practical nor realistic. Furthermore, most of the enormous advances in understanding the pathobiology of PH and the subsequent evidence-based diagnostic and complex treatment algorithms came from the developed world.^1^

It has been estimated that PH is likely to be more prevalent in the developing world (DW) because of the presence of many risk factors affecting millions of people, most of whom cannot get any sort of treatment.^2^ Such risk factors are believed to be more common in the DW compared to the developed world and include infections (such as schistosoma, HIV, viral hepatitis, etc.), uncorrected congenital heart diseases, hemolytic anemias, smoking, and others.^3–11^ Differences in the prevalence of PH, causes, pathobiology, and response to treatment may exist between the developed and developing worlds; hence, it is vital to address these issues directly in the developing countries and not to extrapolate it from the Western literature.

The initiative of addressing the challenges in managing PH in the DW came from recent work between the Pulmonary Vascular Research Institute (PVRI) and the Aswan Heart Center.^12^ The purpose of this article is to address the issue of PH in the DW as a whole and not to provide a specific solution in every single country. We shall explore the prevalence of the PH, address the challenges of PH management, and give practical advice on how to overcome these challenges in the DW. By doing so, we hope to increase awareness, develop successful networks, and help in initiating training and educational programmes for local physicians and medical staff, identifying and establishing local centres of excellence.

All efforts were made to develop this document in an easy-to-read form, making it handy and helpful to clinicians dealing with PH patients in the DW.

**Acknowledging the lack of direct evidence in many recommendations, it is important to reemphasize that this statement is not meant to substitute for international clinical guidelines or the clinicians’ experience or decision.**

## Definition of pulmonary hypertension

Pulmonary hypertension is a hemodynamic and pathophysiological state and not a disease *per se.*^13^ It has been shown to be associated with, and caused by, various clinical conditions that may or may not share similar histological and pathophysiological abnormalities. According to the international guidelines,^14^ PH is classified in to five groups based on similar mechanism, histopathology, clinical pheno- and endo-typing, and response to therapy (Table 1).

**Table 1:** Updated clinical classification of pulmonary hypertension (PH).

**1 Pulmonary arterial hypertension (PAH)**
1.1 Idiopathic PAH
1.2 Heritable PAH
1.3 Drug- and toxin-induced PAH
1.4 PAH associated with:
1.4.1 Connective tissue disease
1.4.2 HIV infection
1.4.3 Portal hypertension
1.4.4 Congenital heart disease
1.4.5 Schistosomiasis
1.5 PAH long-term responders to calcium channel blockers
1.6 PAH with overt features of venous/capillaries (PVOD/PCH) involvement
1.7 Persistent PH of the newborn syndrome
**2 PH due to left heart disease**
2.1 PH due to heart failure with preserved LVEF
2.2 PH due to heart failure with reduced LVEF
2.3 Valvular heart disease
2.4 Congenital/acquired cardiovascular conditions leading to post-capillary PH
**3 PH due to lung diseases and/or hypoxia**
3.1 Obstructive lung disease
3.2 Restrictive lung disease
3.3 Other lung disease with mixed restrictive/obstructive pattern
3.4 Hypoxia without lung disease
3.5 Developmental lung disorders
**4 PH due to pulmonary artery obstructions**
4.1 Chronic thromboembolic PH
4.2 Other pulmonary artery obstructions
**5 PH with unclear and/or multifactorial mechanisms**
5.1 Hematological disorders
5.2 Systemic and metabolic disorders
5.3 Others
5.4 Complex congenital heart disease

PH is generally defined as a mean pulmonary arterial pressure (mPAP) ≥25 mmHg (this definition will probably be changed soon to >20 mmHg) at rest as assessed by right heart catheterization (RHC).^13^ This creates a real problem in the DW because RHC facilities are extremely limited or not available in many situations mandating the need for alternative diagnostic tool. Other hemodynamic values such as pulmonary vascular resistance (PVR), pulmonary artery wedge pressure (PAWP), or cardiac output (CO) are not part of the general definition of PH but are used to subclassify the disease (Table 2). These parameters are also used to characterize the disease, assess severity and response to treatment, or as prognostic measures in managing PH patients.

**Table 2:** Hemodynamic definitions of pulmonary hypertension (PH).

Definitions	Characteristics	Clinical groups#
Pre-capillary PH	mPAP > 25 mmHg PAWP ⩽ 15 mmHg PVR ⩾ 3 WU	1, 3, 4 and 5
Isolated post-capillary PH (IpcPH)	mPAP > 25 mmHg PAWP > 15 mmHg PVR < 3 WU	2 and 5
Combined pre- and post-capillary PH (CpcPH)	mPAP > 25 mmHg PAWP > 15 mmHg PVR ⩾ 3 WU	2 and 5

For the sake of this document, PH will also be defined as right ventricular systolic pressure (RVSP) >35 mmHg on transthoracic echocardiography (ECHO) in the absence of pulmonary stenosis and acute right heart failure (RHF). PH severity is further defined as mild if RVSP is 36–50 mmHg, moderate if RVSP is 51–60 mmHg, and severe if RVSP is >60 mmHg. Subclassification of PH into one the five known subgroups using echocardiography is more challenging, hence, RHC should always be done, whenever available.

## Prevalence of pulmonary hypertension in the developing world

With the exception of a handful of recent registries from Africa and the Middle East, the epidemiology of PH in the DW and the distribution of its etiologies are still largely unknown. Limited reports suggest that the incidence of PH in the DW might be higher than that reported in developed countries because of the prevalence of many conditions that are considered as risk factors for PH such as HIV, hemolytic anemias, schistosomiasis, rheumatic and congenital heart diseases, pulmonary tuberculosis and subsequent lung damage, chronic viral hepatitis and others.^15–19^

In the recent years there has been an increasing awareness of PH in the DW.^20^ This applies to the recent recognition of the importance of PH as a primary disease and as a poor prognostic marker for many other diseases. Unfortunately, the exact estimation of PH prevalence is very difficult in the DW because of extraordinary socio-cultural, ethnical differences, geographic and economic diversity, and substantial regional variations in human development and health care infrastructure. Nevertheless, some data has been published looking at different angles of PH.

Rheumatic heart disease (RHD) remains as one of the common causes of PH in the DW but not in the developed world. Also, it has been estimated that around 33 million patients are currently living with HIV/AIDS, most of them are in DW,^21^^ ^and about 0.5% of them might develop PH.^22,23^ Similarly, schistosomiasis is highly prevalent in Brazil, North Africa, and the Middle East, with an estimation that more than 200 million individuals are infected with this parasite. In one study from Brazil, 30% of pulmonary arterial hypertension (PAH) was caused by schistosomiasis.^24,25^ Finally, an estimated 30 million people suffer from hemoglobinopathies worldwide, most of them reside in the DW. Nearly 10% of this patient population might expect to develop PH.^26^

In Africa, the largest study on PH/RHF showed 697 (28%) of the 2505 patients presented with *de novo* heart failure were diagnosed with PH/RHF between 2006 and 2008.^18^ PH/RHF was the primary diagnosis in 50% of those patients. Most patients presented clinically with dyspnea and found to have a mean RVSP >50 mmHg. Left heart disease (31%), chronic lung disease due to chronic obstructive pulmonary disease and tuberculosis (26%), and PAH (20%) due to HIV, congenital heart diseases (CHD) or idiopathic PAH (IPAH) were the most common causes of PH.

Another study described the prevalence of PH in patients with RHD at a tertiary centre in Nigeria.^27^ Of 1312 ECHO studies, 10% had evidence of rheumatic heart diseases (RHD) and secondary PH was present in 80% of those patients. Another African study found that hypertensive heart disease (25%), peripartum cardiomyopathy (25%), dilated cardiomyopathy (17%) and RHD (13%) are the most common causes of PH.^28^ A similar study from Uganda showed a prevalence of PH in 309 patients presented with newly diagnosed RHD of 33%.^29^ A smaller survey from Lagos, Nigeria, investigated reported a 1% prevalence of PAH in HIV patients presented with cardiovascular diseases.^30^ However, PH with (RVSP > 30 mmHg) was present in 4% of long-term survivors of vertically acquired HIV infection in Zimbabwe.^31^ On the other hand, a screening study of patients with sickle-cell disease (SCD) in Nigeria showed a PH prevalence of 25%,^32^ while a study from Egypt indicates that patients with β-thalassemia might be at increased risk of PH.^33^ Finally, a study from Sudan described 14 consecutive cases of PH with previously treated pulmonary TB.^34^

The Pan African Pulmonary hypertension Cohort (PAPUCO) study is an African registry^20^ and is discussed in detail elsewhere in this special issue by Mocombi et al.

The Egyptian collaborative PH registry has included 260 patients since 2016 with either PAH (190, 73%) or CTEPH (70, 27%) diagnosed with RHC. Patients with congenital heart disease were excluded. The mean age was 35 (±12) years and 69% were females. The mean duration between symptom onset and a proper diagnosis was 39 (±13) months. Most of the patients were already in WHO functional class III or IV at the time of enrollment with a mean 6 MWD of 306(± 120) meters. 81 patients had CMR at the time of enrollment with a mean RVEF 36% (± 13.5%). The most common subgroup of PAH was IPAH (62%). Acute vasoreactivity testing was performed in 139 patients; Only 6% showed positive acute vasoreactivity. PAH-CTD represented only (7%) of PAH patients, however, it could be attributed to referral bias. Only 1% of PAH patients had schistosomiasis. Although most of the patients had advanced disease, only 37% were receiving combination PH-specific therapy at the time of enrollment. Patients with CTEPH were relatively young compared to other registries (mean age was 37 (±10) years). Previous VTE was documented only in 51% of CTEPH patients. 44% of of them underwent pulmonary endarterectomy.

In a hospital-based study from India, a CHD prevalence of 4/1000 live births has been reported, with PDA and VSD being the most common lesions.^35^ A similar prevalence rate was reported by many other population-based studies in the same country.^36–39^ It has been estimated that approximately 200,000 infants with CHD are born every year in India,^40^and another 40,000 cases are diagnosed during childhood.^41^ Furthermore, it has been estimated that 3–10% of infant mortality in India is attributed directly to CHD.^40^ Presently, only 2–3% of infants born with CHD receive timely attention for surgical correction, while the vast majority of the remaining CHD population either fails to survive beyond early childhood or develops irreversible pulmonary vascular disease. At present, 24% of all cardiology admissions in India are related to CHD.^42^

Schistosomiasis is a major health concern in many countries in the Middle East, Africa, China, and Latin American countries, especially Brazil.^43–50^ In a single center at the north-eastern region of Brazil, schistosomiasis was the most prevalent etiology (39%), followed by PAH-CHD (26.9%), IPAH (14.6%), and PAH-CTD (12.2%).^51^

In China, it has been estimated that there are 2,500 to 5,000 new IPAH patients each year and a much higher number for other forms of PH.^52^ In Iran, the IPAH registry was opened in November 2009. One hundred and sixteen patients (82 females, 34 males) were included. The mean age was 36.69 ±14 years. Of those, approximately 8% were in New York Heart Association (NYHA) functional class IV, 53% class III, 37% class II and 2% class I. Dyspnea on exertion was the most common symptoms (98%), followed by extreme fatigue (92%), chest pain (56%) and syncope (34%).^53^

Finally, a report of 107 incident PAH patients (diagnosed by RHC) in Saudi Arabia was published.^54^ The mean age at diagnosis was 36 (±9) years, with a female preponderance of 62.6%. The mean duration between symptom onset and diagnosis was unacceptably long (27.8 ± 9.0 months). As a result, 72.8% of the patients were in functional class III or IV at presentation. IPAH was the most common subtype of PAH. CHD was the second most common PAH subgroup and had the best physiological parameters compared to other groups.

Such high prevalence of PAH-CHD in Saudi Arabia probably reflects the current practice of late detection of CHD patients and delayed surgical corrections. Systemic sclerosis (SSc) was the leading cause of PAH-CTD. Heritable and portopulmonary PAH were the least common causes and had the worse outcome. None of the patients tested positive for schistosoma serology, however, this might be attributed to detection or referral bias. Only 5 patients (4.7%) were candidate to use calcium channel blockers based on reversibility testing. Thirty-one patients (28.9%) died during the 3-year follow up period.^55^

Another report from Saudi Arabia described the distribution of PH in 112 patients diagnosed by RHC found that 10.7% belonged to PAH, 6% to PH due to left heart disease, 65% to PH due to lung diseases, 3.5% to chronic thromboembolic PH (CTEPH), and 14.2% related to WHO group V diseases.^56^ However, this center is recognised as the national center of interstitial lung disease and referral bias can explain the high prevalence of PH due to lung disease in this cohort.

## Challenges for PH management in the developing world

### 1. Challenges in clinical diagnosis

The median duration of symptoms before establishing the diagnosis in the developed world is more than two years.^57–59^ In developing countries with limited access to adequate health care services, the duration of symptoms is likely to be much longer.^54^ In many situations, both the patients and the physicians tend to miss the diagnosis, especially with the paucity and subtlety of physical signs in early disease. Breathlessness is often attributed to other causes like asthma or even considered psychogenic in origin. Syncope is often considered to be neurogenic or psychogenic. Cultural beliefs and practices, as well as socio-economic factors may delay medical consultation. Physicians with limited awareness and facilities to investigate may further add to the problem. The financial burden may also force some patients to go towards traditional cheaper approach and further delay the diagnosis. Lastly, governmental healthcare providers may often have little interest in supporting PH related educational and treatment programs as the disease is still considered rare, especially if the country is experiencing other large-scale health threats.

### 2. Challenges in diagnostic evaluation

#### Initial evaluation

Although that both ECG and CXR might be abnormal in advanced disease, they are usually not specific in early stages.

#### Echocardiography

Two-dimensional (2D)-echocardiogram can be an ideal screening tool but has its own limitations as a diagnostic strategy,^13^ as the findings may be subtle in early disease and an inexperienced operator can easily miss the diagnosis on routine echocardiography exam. Under- or over-diagnosis of PH is possible and is troublesome.^60^ Furthermore, echocardiography has its limitation in identifying a specific cause or differentiating between different groups of PH (e.g., pulmonary arterial hypertension (PAH) versus heart failure with preserved ejection fraction (HFpEF). Also, echocardiography cannot provide reliable values for pulmonary vascular resistance (PVR), pulmonary artery wedge pressure (PAWP) or cardiac output (CO).^61–67^

Nevertheless, Doppler echocardiography proved to be a reliable method for the estimation of sPAP, being well suited to establish the non-invasive diagnosis of PH in patients with cardiac diseases.^68^ The estimation of sPAP has shown a good correlation with RHC and is based on the peak velocity of the jet of tricuspid regurgitation. Despite its limitations, echocardiography is not readily available in many centers within the DW.

#### Right heart catheterization

Right heart catheterization is the gold standard tool to diagnose PH and should always be for this purpose, whenever available.^13^ The safety of this procedure in experienced hands has been well established.^69^ RHC is only available in a very few centers in the DW. Availability and cost are the important considerations in many developing countries where resources are limited. Furthermore, lack of experience and unfounded fear of complications are other reasons for avoiding RHC.

### 3. Challenges in medical therapy

In many developing countries, PAH-specific therapy is considered too expensive and not readily available. For economic restrictions, insurance and governmental agencies may not be able to sponsor or reimburse the costs of the medications. Unfortunately, despite high demand, major drug companies are not currently interested in these poor markets, and it seems there are no plans in the near future to reduce the cost for PH patients in these countries. On many occasions, generic medications are partially available and are commonly used at sub-optimal doses to save money. Some physicians mistakenly treat right heart failure due to PH with beta-blockers and angiotensin converting enzyme inhibitors in a similar way they treat left heart failure. Other physicians use calcium channel blocker as empiric therapy without RHC study or determining the vasoreactivity. Both can be detrimental to the patients and raise again the inadequate knowledge and awareness of the disease among health care providers.

### 4. Challenges in surgical treatment

Lung transplantation is not available in most DW (with a few exceptions, such as India and Saudi Arabia). The facilities and expertise for pulmonary endarterectomy are not available in most countries of the DW. Development of facilities for these procedures is complex and beyond the physicians’ domain.

The authors recommend six main strategies/phases to overcome these challenges. However, these phases may run in parallel in certain situation and not necessarily sequential.

#### Phase I: Collecting data and establishing the infrastructure

The idea is not to provide a formal registry, but rather to provide some form of a data collection concerning the disease from both active patients and the patients-at risk. A standard database format may be established by the PVRI via a specific taskforce. This taskforce should liaise with health care providers and the responsible authorities in the developing countries/regions and monitor work progress. It is recommended for this database to be available online, whenever applicable, to facilitate data sharing between centres in the DW and the PVRI taskforce. We might also look towards the PAPUCO experience as a possible example for this database. The PAPUCO project originated in South Africa and involved different countries on the continent and has overall been very successful.^20^

As different developing countries might have different health care systems, economic power and/or specific needs, creating a regional map that categorizes each country into specific sections/zones might be necessary. Therefore, as previously outlined, a later stage of the project will include developing of the PVRI consensus statement for establishing country’s specific guidelines in order to meet the needs of each country/zone. This can be achieved by having multiple small cross-sectional surveys in different regions of the DW.

#### Phase II: Education

The educational process has many tasks that include:

**Specific awareness**: The core of the educational task is *to establish the specific awareness* plan for each country, to support the selection of the different leaders in each country/zone, and to provide an extensive training program for the leaders. This includes establishing specific “masterclass” courses and various PH symposia designed to educate the individual country/zone leaders. The courses and symposia should be facilitated by experts in the field and organised by the assigned taskforce. The establishment of the Saudi Association of Pulmonary hypertension and its great success in promoting awareness and education of different aspects of PH in the Arab countries can be used as a role model to be used in other countries in the DW.^70–86^

**General awareness:** This is usually achieved by running many awareness days and workshops that include general knowledge about the presentation of the disease, risk factors, and prognosis. It should also include the referral mechanism. This activity should be adjusted to suit physicians (both specialized and general), nurses, patients, media, and authorities. The PVRI can help by producing a generic template for a leaflet/booklet, which can then be further detailed adhering to the needs of the specific country/region.

**Maintaining awareness is key for success:** The message should be consistent and repetitive via continuous training for different ranks of doctors (junior and senior doctors alike), nurses, and other healthcare providers.

### Establishing telemedicine and teleconferences program and virtual consultation service with experts in the PVRI.

**Holding annual regional PH symposia to share experience.** World experts should be invited to these symposia to disseminate knowledge and information. They may also be involved in direct patient consultation.

#### Phase III: Centers of Excellence

Creating the ‘centres of excellence’ for each country/region is of great importance to take this project to a higher level. These centres are to serve for both management and training. Specific country/region-based criteria should be developed for each region. A measure for PVRI-accreditation system for these centres should be established to ensure quality.

These centres may provide following tasks:

• Establishment of referral criteria
• Basic diagnostic tools (CXR, ECG, ECHO, CT scan, PFT lab)
• Able to perform or refer patients for RHC
• Cardiology and pulmonary services
• Medical intensive care services
• Basic research facility to help PH researchers in their own countries, and to put them in touch with the centres of excellence in the developed countries.

A research project may be addressed in these settings which could prove very useful to that particular country/region; e.g., high-altitude PH or PH secondary to infectious diseases. Contributing to phase III international clinical trials may also be possible in selected centres.

However, in some developing countries with very limited resources it might be more practical to establish “centres of experience”, where all new PH patients should be referred to in order to build adequate clinical experiences. The criteria for such centers are:

• Establishment of referral criteria
• Basic diagnostic tools (CXR, ECG, ECHO)
• Cardiology and pulmonary services
• Medical intensive care services

#### Phase IV: Diagnostic and treatment algorithms

Development of “unique” diagnostic and treatment algorithms based on realistic situations and available resources. Such algorithms should include the minimal acceptable criteria to suite all developing countries and should be amended by each country/region to meet its needs. These algorithms might not be evidence-based at the early stage and might include many “expert opinion” recommendations. However, in future, these recommendations should be validated based on real data obtained from each country.

#### Phase V: Funding:

Certainly, this is the cornerstone of the project. Fundraising should be tried on many levels and should include:

• Local fundraising
• Involving local governments and authorities
• Directing drug companies to the DW’s “market”
• International charity programs
• PVRI direct funding for certain research projects

The fund should be directed towards different fields that include:

• Purchasing basic equipment to the peripheral clinic, which serve as the potential case-finding. Such equipment may include CXR and ECG machines. More sophisticated equipment such as ECHO, V/Q scanner, CT scan, cardiac catheters, etc. are also needed in the selected centres of excellence
• Sponsoring educational activities, such as the awareness courses, the masterclasses, international symposia, public awareness and media coverage
• Help in funding the medication (the presence of generic alternatives might help in reducing the cost in these countries).

#### Phase VI: Patient associations

Although this is not a formal PVRI task, it is still recommended that the final consensus paper should indicate the basic points of reference to set up patients’ associations in specific countries/regions. This is important as it will help with future fundraising, case studies, and promotional materials.

In conclusion, the aim of this project is to improve the field of PH management in the DW, acknowledging the difficulties and the challenges mentioned in this paper. The effort should be focused on the work for improving awareness and education, collecting date, and establishing of regional referral centers, and to optimize resources to give the best evidence-based medicine with better patient outcomes. The problems facing the management of PH in the DW may be summarized as:

**Table 3:** Recommendation for clinical evaluation (all patients).

Recommendation	Class of recommendation	Level of Evidence
Detailed history has to be obtained in all patients to understand the nature and severity of symptoms.	I	C
WHO Functional class of the patients’ symptoms has to be established.	I	C
Meticulous clinical examination has to be done in all patients to establish the diagnosis of PAH.	I	C
Diligent search should be made in history and examination to rule out secondary causes of PH	I	C
All patients with PH should have a baseline 6MWT done.	I	C
All patients with PH should have a baseline ECG	I	C
All patients with PH should have a baseline CXR	I	C

**Notes.**

WHO FC world health organization functional class PH pulmonary hypertension 6MWT six-minute walk test ECG electrocardiogram CXR chest X ray

**Table 4:** Echocardiography protocol for the diagnosis of pulmonary hypertension (if available).

Parameters	Comments
Assess LV systolic & diastolic function	Rule out group 2 diseases
Assess MV & AV structure and function	Rule out rheumatic heart disease
Assess LA dimensions & area	Central or rightward position of the inter-atrial septum indicates a left atrial pressure higher than or equal to the right atrial pressure and makes the diagnosis of PAH less likely.
Assess RV & RA dimensions and function	∘ RV free wall thickness ∘ RV dimensions ∘ RA volume index ∘ TAPSEo Tei index
Assess PV & TV structure and function	Rule out RHD
Exclude RVOT obstruction	PS [valvular /sub-valvular]
Measure TR	Best possible jet/Doppler alignment, use contrast if necessary
To assess for intra-cardiac shunts	Use contrast if needed
IVC collapsibility	For the estimation of RAP

**Notes.**

LV left ventricle MV mitral valve AV aortic valve LA left atrium RV right ventricle RA right atrium PV pulmonary valve TV tricuspid valve RVOT right ventricular outflow tract TR tricuspid regurgitation IVC inferior vana cava PAH pulmonary arterial hypertension TAPSE Tricuspid annular plain systolic excursion RHD rheumatic heart disease PS pulmonary stenosis RAP right atrial pressure

**Table 5:** Echocardiographic criteria for screening of high-risk patients for pulmonary hypertension, if available.

Likelihood for PH	Criteria	Level of Evidence
PH unlikely	TRV ≤ 2.8 m/s, and sPAP ≤ 35 mmHg, and No additional echocardiographic criteria for PH, and Asymptomatic	B
PH possible	***Criteria A*** • TRV ≤ 2.8 m/s, and • sPAP ≤ 35 mmHg, and	C
	• Presence of additional echocardiographic criteria for PH, or symptoms suggestive for PH ***Criteria B*** • TRV 2.9 - 3.4 m/s, or • sPAP 36 - 45 mmHg	C
PH likely	• TRV > 3.4 m/s, or • sPAP > 45 mmHg	B

**Notes.**

PH pulmonary hypertension TRV tricuspid regurgitation jet velocity sPAP systolic pulmonary artery pressure RHC right heart catheterization PAH pulmonary arterial hypertension. (Modified from Idrees et al, with permission).^70^

**Table 6:** Hemodynamic parameters measured during RHC, if available.

Parameter	Class of recommendation	Remarks
RAP	I	
CO/CI	IIa (see remarks)	By thermodilution (or by the Fick method in cases of systemic-to-pulmonary shunts)
MvO_**2**_%	IIa	
PVR	I	Needed for the diagnosis of PAH
mPAP	See remarks	For diagnostic purpose: Class of recommendation I
		For prognostic purpose: Class of recommendation IIb
PAWP	I (see remarks)	In case of inaccurate wedging, LVEDP should be measured
Vasoreactivity	See remarks	Class of recommendation I in IPAH
		Class of recommendation IIa in CHD-PAH and CTEPH
		Class of recommendation IIb in other forms of PH

**Notes.**

RAP right atrial pressure CO/CI cardiac output/cardiac index MvO2% mixed venous oxygen saturation PVR pulmonary vascular resistance mPAP mean pulmonary artery pressure PAWP pulmonary artery wedge pressure

1. The size of the problem: Very limited data is available on the prevalence and the extent of the problem in the DW. However, it is likely that the magnitude of the problem might be enormous given that many diseases known to be associated with PH, such as uncorrected congenital heart diseases, schistosomiasis, HIV, rheumatic heart diseases, hemolytic anemia, and parenchymal and chronic pulmonary airway disease are prevalent in developing countries.
2. Different developing countries might have different problems, needs, and resources, and so local guidelines should be established to meet the basic or minimal criteria needed to build a clinically meaningful PH program.
3. There are potential differences in etiology of PH between developing and developed countries. Epidemiological data regarding PH and disease-specific registry data is available from many developed countries but not from developing countries. The spectrum of the diseases causing PH is likely to be different between the developed and developing worlds. PH due to CHD, RHD, schistosomiasis, hemolytic diseases, and HIV infection are more important causes for PH in the developing countries, but they are much less common in the developed countries where biological research or drug development takes place. Furthermore, clinical trials done to test new drugs may exclude the typical PH patients of the developing countries. By extrapolation, PAH-specific drugs are used in these conditions, though their efficacy cannot be certain.
4. Poor awareness about the disease and its burdens between patients, health care providers, and the authorities
5. One of the most difficult problems to overcome is the weak economic power, which is present in most developing countries, and the absence of the basic infrastructure and medical resources required to diagnose and treat PH patients. Such economic challenges make recommendations concerning evidence-based diagnostic algorithm given by international (Western) guidelines unrealistic and inapplicable. The authors have suggested general recommendation concerning diagnosis and management of PH in the DW (Tables 3–6).
6. The controversy about the ownership of PH project can be a major issue that may halt the whole project. In many developing countries, certain parties try to own such projects to gain power, including political, financial or medical. Hence, it is very important to keep the administrative process of this project under the PVRI, who should collaborate with certain “representatives”/task leaders in each country/region.

## The way forward

There are many knowledgeable and competent physicians in the developing countries, who are interested in PH management. They should work together in their respective regions and strive to educate the other physicians in early diagnosis of PH. A global project is urgently needed in order to improve the outcome of PH in the DW, acknowledging the difficulties and the challenges mentioned in this paper.

The international institutes, such as the PVTI, should work with the local initiatives in the DW to improve awareness and education, collecting data, establishing regional referral centers, and focusing on optimizing resources in order to build collaborative PH programs in the DW.
